# Targeted enhancement strategies for Sojae Semen Praeparatum: Impact of *Aspergillus oryzae* and *Bacillus subtilis* on microbial communities, flavor substances, and functional components

**DOI:** 10.1016/j.fochx.2025.102931

**Published:** 2025-08-21

**Authors:** Bin Wang, Tianxing He, Yingying Cheng, Hongping Chen, Yuan Hu, Youping Liu, Fu Wang, Lin Chen

**Affiliations:** State Key Laboratory of Southwestern Chinese Medicine Resources, Department of Pharmacy, Chengdu University of TCM, Chengdu, Sichuan, China

**Keywords:** Sojae Semen Praeparatum, enhancement fermentation, flavor substances, functional components, core functional microorganisms

## Abstract

Sojae Semen Praeparatum (SSP), a traditional Chinese fermented soybean product, was optimized through novel single/double enhancement fermentation using *Aspergillus oryzae* and *Bacillus subtilis*. Enhanced fermentation significantly increases the production of flavor amino acids. Using headspace solid-phase extraction microextraction gas chromatography-mass spectrometry (HS-SPEM-GC-MS) technology, 51 critical flavor substances were identified, confirming that enhanced fermentation improves the flavor profile of SSP. Isoflavone quantification revealed that enhancement strategies promoted isoflavone conversion. Enzyme inhibition and antioxidant activities were superior in the double enhancement fermentation group. Liquid chromatography-tandem mass spectrometry (LC-MS/MS) analysis demonstrated a significant increase in SSP's enrichment in the flavone and flavonol biosynthesis pathway. Screening identified 19 key flavonoid components strongly correlated with bioactivity, and enhancement fermentation notably enhancing their accumulation. Metagenomic sequencing revealed 14 key differential microorganisms, essential to flavor development and activity enhancement in SSP. This study offers valuable insights for optimizing fermentation processes to enhance product quality.

## Highlights

• Study presents single/double enhancement fermentation for SSP.
• Double enhancement boosts flavor amino acids and identifies 51 flavor compounds.
• Superior enzyme inhibition and antioxidant activities in double enhancement group.
• LC-MS/MS shows increased flavonoid biosynthesis with double enhancement.
• Metagenomics reveal 14 key microorganisms vital for flavor and activity.

## Graphical abstract

**Figure:**
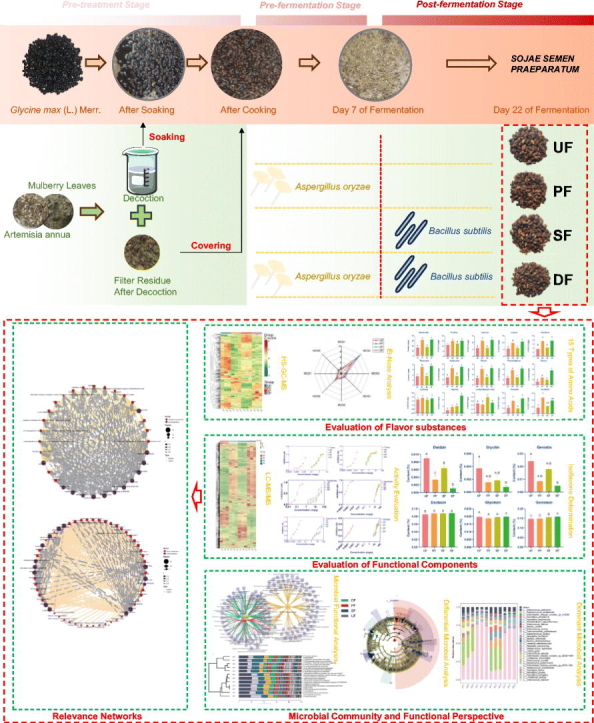

## 1 Introduction

Sojae Semen Praeparatum (SSP), a traditional Chinese medicinal fermented soybean product, has garnered considerable attention for its unique flavor and potential health benefits, including antidepressant effects, blood glucose regulation, lipid-lowering properties, and antioxidant activity (Chen et al., 2021; Ge et al., 2006; Liu, Huang, et al., 2023; Luo et al., 2021; Wang, Cheng, et al., 2024). Major production regions in China include Sichuan, Jiangxi, Hebei, Guangdong, and Anhui (Li, Li, et al., 2024; Xie et al., 2023). SSP production involves several critical steps—soaking, steaming, and fermentation—which are critical to the development of its characteristic flavor compounds and bioactive components (Chai et al., 2019). Among these, fermentation, driven by a diverse array of microorganisms, plays a pivotal role in shaping SSP’s flavor and functionality (Wang, Shi, et al., 2024). Traditional SSP fermentation comprises two stages: an open-environment pre-fermentation and a closed-environment post-fermentation. The complexity of this process and the challenges in controlling microbial sources lead to substantial quality variations across different regions, batches, and seasons (Guo et al., 2018). This variability underscores the need to optimize the traditional fermentation process. Modern fermentation techniques utilizing inoculated starters have been widely adopted in fermented food production to enhance product quality and ensure safety by controlling microbial contamination (Nie et al., 2024). Such techniques have been successfully applied to various traditional foods, such as rice noodles (Huang et al., 2024), baijiu (Tong et al., 2024), and meat products (Franciosa et al., 2022). However, the distinct two-stage fermentation process of SSP differs from that of typical fermented soybeans, making single-inoculation microorganisms susceptible to environmental fluctuations between stages (Wang et al., 2024). This study, therefore, proposes a targeted enhancement fermentation strategy for SSP, incorporating both single enhancement fermentation (focusing on either the pre- or post-fermentation stage) and double enhancement fermentation (enhancing both stages). To date, no comprehensive study has investigated the effects and underlying mechanisms of this targeted enhancement strategy on SSP quality.

The yellow spores of *Aspergillus* are ubiquitous in the fermentation substrate and serve as a key indicator marking the completion of the pre-fermentation stage of SSP (Chinese Pharmacopoeia Commission, 2020). *Aspergillus oryzae*, a filamentous fungus, plays a critical role in the fermentation of various East Asian foods, with a history of over 1,000 years in Daqu preparation (Seidler et al., 2024). Fermentation with *A. oryzae* contributes to the production of organic acids and isoflavones in legumes, enhancing flavor and boosting antioxidant activity (Allwood et al., 2021; Chiou & Cheng, 2001; Lee et al., 2022). In a previous study, *A. oryzae* was identified as a core functional microorganism in the pre-fermentation stage of SSP, where it improves both flavor and functionality by increasing amino acids such as serine and lysine, as well as isoflavones like daidzein and genistein (Wang et al., 2024). In contrast, the post-fermentation stage plays a critical role in developing SSP's distinctive flavor profile. The characteristic aroma produced during this stage signals the completion of post-fermentation (Chinese Pharmacopoeia Commission, 2020). Among the various microorganisms involved in the post-fermentation of SSP, *Bacillus subtilis* is particularly important for promoting the formation of the unique aroma and enhancing the content of γ-aminobutyric acid (GABA) (Xiong et al., 2019; Xu et al., 2022). Additionally, fermentation with *B. subtilis* imparts a soy sauce-like aroma (Tan et al., 2024), increases peptide and free amino acid levels (Gao et al., 2024), and significantly enhances antioxidant properties (Dai et al., 2024). The inoculation of *A. oryzae* and *B. subtilis* in soybean fermentation has become a widely adopted model (Hyeon et al., 2020; Shukla et al., 2015). Therefore, this study selects *A. oryzae* and *B. subtilis* as the inoculated microorganisms for the enhanced fermentation of SSP. It was hypothesized that the combined use of single and dual-culture will synergistically enhance the flavor and bioactive compounds in SSP, leading to superior fermentation outcomes.

Recent advancements in metagenomic technology have provided a comprehensive and effective approach to understanding microbial composition. Its exceptional gene annotation capabilities offer distinct advantages in identifying functional microorganisms and elucidating their metabolic pathways (Li, Cao, et al., 2024). This cutting-edge technology has been extensively applied to analyze key microorganisms and discover functional traits in fermented soybean products (Han et al., 2024; Kharnaior & Tamang, 2023; Wicaksono et al., 2024). Simultaneously, metabolomics has emerged as a crucial tool for investigating microbial metabolic transformations at the metabolite level (Wang, Hu, et al., 2023), complementing the broader understanding of the metabolic roles of key microorganisms. Metabolomics focuses on the qualitative and quantitative analysis of small-molecule metabolites (< 1,000-1,500 Da), allowing for the comparison of sample variations (Mahato et al., 2024). Analytical platforms such as nuclear magnetic resonance (NMR), gas chromatography-mass spectrometry (GC-MS), liquid chromatography-mass spectrometry (LC-MS), and capillary electrophoresis-mass spectrometry (CE-MS) are (Liu et al., 2023). widely used in metabolomic studies. Among these, GC-MS and LC-MS are frequently employed to profile both volatile and non-volatile metabolites in fermented soybean products (Lee et al., 2021). Additionally, solid-phase microextraction-headspace sampling (SPME-HS), a highly efficient, non-destructive, and convenient sample preparation method, is often paired with GC-MS for an in-depth analysis of aromatic metabolites in SSP (Guo et al., 2024). When combined with metagenomics, metabolomics enables a multidimensional evaluation of sample quality, encompassing metabolic transformations and microbial functions, and uncovers the mechanisms underlying quality formation. The integration of metabolomics and metagenomics thus represents a powerful strategy for screening core functional microorganisms and identifying key metabolites.

This study introduced a targeted enhancement fermentation strategy for SSP, focusing on its effects on quality and underlying mechanisms. HPLC-DAD was employed to detect flavor amino acids, followed by electronic nose (E-nose), combined with HS-SPME-GC-MS for comprehensive flavor characterization. Key isoflavone components were quantified. LC-MS/MS, in conjunction with *in vitro* antioxidant and enzyme inhibition assays, identified functional components. Metagenomic analysis assessed microbial community dynamics and core microorganisms. Finally, correlation analysis was performed to investigate comprehensively the impact of various fermentation strategies on the microbial communities, flavor substances, and functional components, ultimately aiming to enhance SSP quality. This research was the first to integrate multi-omics technologies to evaluate these effects, providing valuable insights for optimizing SSP quality.

## 2 Materials and Methods

### 2.1 Materials

SSP was prepared using various enhancement fermentation strategies at a single location in Sichuan, China. The preparation followed established protocols from previous research (Wang et al., 2024) and the methods outlined (Chinese Pharmacopoeia Commission, 2020). Black beans were soaked overnight in filtrates of mulberry leaf and *Artemisia annua* and then steamed. Pre-fermentation was initiated, during which yellow mycelium appeared on the surface of the sample after 7 days. The samples were then washed and subjected to post-fermentation in sealed containers, resulting in unenhanced fermentation samples (UF) after 22 days of total fermentation. For enhanced pre-fermentation (PF), a spore suspension of *A. oryzae* (5% (v/m), 1 × 10^7^ CFU/mL) was inoculated immediately after steaming, followed by natural fermentation until day 22. Enhanced post-fermentation (SF) samples were washed after 7 days of pre-fermentation, then inoculated with a *B. subtilis* suspension (5% (v/m), 1 × 10^7^ CFU/mL), followed by natural fermentation until day 22. The double-enhanced fermentation samples (DF) involved inoculating *A. oryzae* immediately after steaming, followed by washing on day 7 and inoculating *B. subtilis* on the same day (day 7), then continuing natural fermentation until day 22. All fermentation processes were conducted at 30±2°C and 80% humidity. Processed samples were freeze-dried, homogenized by grinding, and sieved (80-mesh). Powdered aliquots were stored at -80°C prior to analysis.

### 2.2 UV measurement of total isoflavone using spectrophotometry

The total isoflavone content was measured following previously established methods (Jiang, Gao, Zhang, Ou and Zhou, 2023; Wang, Shi, et al., 2024). Briefly, 0.25 g of the sample powder was accurately weighed, mixed with 10 mL of methanol, and sonicated for 2 h. After cooling, the weight was recorded, and methanol was added to compensate for any loss. The solution was then filtered through a 0.45 μm membrane. 0.5 mL of the filtrate was placed into a 10-mL volumetric flask with methanol and diluted. The total isoflavone content was determined by measuring the absorbance at 260 nm, using daidzein as the standard compound. Calibration curves with linear ranges are provided in Table S1.

### 2.3 HPLC-DAD measurement of isoflavones

The six specific isoflavones were measured following previously established methods (Jiang et al., 2023). The extraction procedure was identical to that described for total isoflavone analysis. Separation was achieved using an Agilent ZORBAX SB-C18 column (4.6 mm × 250 mm, 5 μm) on an Agilent 1260 series HPLC system equipped with a DAD detector. The mobile phase consisted of (A) 0.2% acetic acid in H_2_O and (B) MeOH. The gradient elution program was as follows: 0-6 min, 20% B; 6-7 min, 20-40% B; 7-12 min, 40% B; 12-22 min, 40-60% B; 22-27 min, 60% B; 27-35 min, 60-90% B; 35-40 min, 90% B; 40-41 min, 90-20% B; 41-45 min, 20% B. The flow rate was 1.0 mL/min, the column temperature was maintained at 30 °C, and the detection wavelength was set at 260 nm. The injection volume was 10 μL. Calibration curves with linear ranges for each isoflavone are provided in Table S1.

### 2.4 HPLC-DAD measurement of amino acids

The amino acid content was determined using a pre-column derivatization method with phenyl isothiocyanate as described in prior research (Wang et al., 2024; Wu et al., 2023). 100 mg of samples were dissolved in 4.0 mL of 0.1 M HCl, followed by sonication for 20 min. After cooling, samples were centrifuged at 12,000 rpm for 10 min. A 0.5 mL aliquot of the filtrate was mixed in a micro tube with 0.25 mL of 1 M triethylamine and 0.25 mL of 0.1 M phenyl isothiocyanate. The mixture was incubated at 40°C in the dark for 1 h. Then, 1 mL n-hexane was added. After standing for 20 min, the lower clear layer was analyzed by Agilent ZORBAX SB-C18 column (4.6 mm × 250 mm, 5 μm) on an Agilent 1260 series HPLC system equipped with a DAD detector. Mobile phase: (A) ACN: H_2_O (80:20), (B) 0.1 M sodium acetate: ACN (94:6); injection volume: 5 μL; flow rate: 1 mL/min. Linear gradient: 0-20 min, 0% A; 20-28 min, 0–12% A; 28-45 min, 12-25% A; 45-50 min, 25-35% A; 50-55 min, 35-100% A; 55-60 min, 100% A; 60-65 min, 100-0% A. Detection was at 254 nm. Calibration curves with linear ranges for each isoflavone are provided in Table S2.

### 2.5 Electronic nose testing

Electronic nose testing was conducted according to previously reported methods (Wang et al., 2023). An 8 MEMS-MOS E-nose (Isensortalk Co., Ltd., Beijing, China) was employed. 2.0 g of the sample powder was placed into a 20 mL headspace vial for testing. Data acquisition commenced after 60 s of injection to ensure signal stability. Sensor array data from 55-58 s were used for statistical analysis. Post-analysis, the system was purged with filtered clean air for 120 s to re-establish the baseline.

### 2.6 HS-GC-MS analysis of volatile metabolites

#### 2.6.1 Sample preparation and extraction

Referencing previous study (Wang, Li, et al., 2023), Samples were ground to a powder in liquid nitrogen. 0.2 g of the powder was transferred immediately to a 20 mL headspace vial (Agilent), containing 0.2 g NaCl powder and 20 μL of internal standard solution (3-Hexanone-2,2,4,4-d4, 10 μg/mL), to inhibit any enzyme reaction. The vials were sealed using crimp-top caps with TFE-silicone headspace septa (Agilent). At the time of SPME analysis, each vial was placed in 60 °C for 5 min, then a 120 μm DVB/CAR/PDMS extraction head (Agilent) was exposed to the headspace of the sample for 15 min at 60 °C.

#### 2.6.2 GC-MS conditions

After sampling, desorption of the volatile organic compounds (VOCs) from the fibre coating was carried out in the injection port of the GC apparatus (Model 8890; Agilent) at 250°C for 5 min in the splitless mode. The identification and quantification of VOCs was carried out using an Agilent Model 8890 GC and a 7000D mass spectrometer (Agilent), equipped with a DB-5MS capillary column (30 m × 0.25 mm × 0.25 μm, 5% phenyl-polymethylsiloxane; Agilent J&W Scientific). Helium was used as the carrier gas at a linear velocity of 1.2 mL/min. The oven temperature was programmed from 40°C, holding for 3.5 min, increasing at 10°C/min to 100°C, at 7°C/min to 180°C, at 25°C/min to 280°C, hold for 5 min. Mass spectra was recorded in electron impact (EI) ionisation mode at 70 eV. The quadrupole mass detector, ion source and transfer line temperatures were set, respectively, at 150, 230 and 280°C. The MS was selected ion monitoring (SIM) mode was used for the identification and quantification of analytes (Wang, Li, et al., 2023).

#### 2.6.3 Metabolite Qualitative and Quantitative Principles

Based on multiple species, literature, partial standards, and retention indices, a database was established that includes determined retention times (RT) and qualitative and quantitative ions for selected ion detection modes. Each compound is assigned one quantitative ion and 2 to 3 qualitative ions. Ions in each group are detected in chronological order. If the detected retention time matches the standard reference and the selected ions appear in the sample mass spectrum after background subtraction, the substance is identified (Yuan et al., 2022). Quantitative ions are then integrated and calibrated to enhance quantification accuracy.

#### 2.6.4 rOAV Analysis

The relative odor activity value (rOAV) analysis was conducted following relevant literature (Zhang et al., 2025), and the calculation formula is as follows

rOAVi=Ci/Ti

Where rOAVi is the relative odor activity value of compound i. Ci is the relative concentration of the compound (μg/g or μg/mL); and Ti is the threshold of the compound (Threshold, μg/g or μg/mL).

### 2.7 Antioxidant activity and enzyme inhibition rate experiments

The extraction of SSP samples and assessment of antioxidant activities (ABTS, DPPH, and FRAP) were performed according to previously established (Hu et al., 2025; Wang, Hu, Chen, Chen and Liu, 2023; Wang, Wang, et al., 2024) protocols.

Acetylcholinesterase (AChE) inhibitory activity was determined using an enhanced Ellman’s colorimetric method (Shabbir et al., 2022). In a 96-well plate, 20 μL of the sample at various concentrations was mixed with 20 μL of 0.2 U/mL electric eel AChE solution in 150 μL of 100 mM sodium phosphate buffer (pH 7.2) and incubated at room temperature for 15 minutes. Subsequently, 10 μL of 2 mM DTNB and 20 μL of 15.0 mM thioacetylcholine iodide were added. The reaction was allowed to proceed for 30 minutes, and the absorbance was measured at 412 nm, with galantamine serving as the positive control.

α-Amylase inhibitory activity was evaluated using a modified DNS colorimetric method (Lei et al., 2024). Specifically, 20 μL of α-amylase (8 U/mL, PBS) and 20 μL of sample at different concentrations were added to a 1.5-mL centrifuge tube and incubated in a 37°C water bath for 10 minutes. Then, 40 μL of soluble starch (1%, w/v) was added, and incubation continued for an additional 10 minutes. Following this, 100 μL of DNS reagent was added, and the mixture was heated in a boiling water bath for 5 minutes, then rapidly cooled. A 30 μL aliquot of the reaction mixture was diluted, and absorbance was measured at 540 nm, with acarbose as the positive control.

For α-glucosidase inhibitory activity, the assay was performed based on previous research with modifications (Ren et al., 2024). In a 96-well plate, 20 μL of α-glucosidase (1 U/mL, PBS) and 10 μL of the sample were added. After 10 minutes of incubation at 37°C, 30 μL of 10 mM PNPG substrate was introduced, and the reaction was incubated for an additional 30 minutes. The reaction was terminated by adding 80 μL of 1 M Na_2_CO_3_, and absorbance was measured at 405 nm, with acarbose as the positive control.

Pancreatic lipase inhibitory activity was assessed using the modified p-NPP method (Haddou et al., 2023). p-NPP was dissolved in acetonitrile to prepare a 10 mM stock solution, which was then diluted to 3.33 mM. In a 96-well plate, 20 μL of the sample, 10 μL of 3.33 mM p-NPP, 20 μL of 5 mg/mL pancreatic lipase solution, and 150 μL of Tris-HCl buffer were added sequentially. After incubation at 37°C for 60 minutes, the absorbance was measured at 405 nm, with orlistat as the positive control.

### 2.8 LC-MS analysis of non-volatile metabolites

#### 2.8.1 Metabolites extract

Referencing previous study (Sui et al., 2022), The 20 mg samples were taken and lyophilized, mixed with beads and 1,000 μL of extraction solution (MeOH : ACN : H_2_O, 2 : 2 : 1 (v/v)). The extraction solution contains deuterated internal standards. The mixed solution was vortexed for 30 s. Then the mixed samples were homogenized (35 Hz, 4 min) and sonicated for 5 min in 4 °C water bath, the step repeat for three times. The samples were incubated for 1 h at -40 °C to precipitate proteins. Then the samples ware centrifuged at 12,000 rpm (RCF = 13,800 (×g), R = 8.6 cm) for 15 min at 4 °C. The supernatant was transferred to a fresh glass vial for analysis. The quality control (QC) sample was prepared by mixing an equal aliquot of the supernatant of sample.

#### 2.8.2 LC-MS/MS analysis

For polar metabolites (Sui et al., 2022), LC-MS/MS analyses were performed using an UHPLC system (Vanquish, Thermo Fisher Scientific) with a Waters ACQUITY UPLC BEH Amide (2.1 mm × 50 mm, 1.7 μm) coupled to Orbitrap Exploris 120 mass spectrometer (Orbitrap MS, Thermo). The mobile phase consisted of 25 mM ammonium acetate and 25 ammonia hydroxide in water (pH = 9.75) (A) and acetonitrile (B). The auto-sampler temperature was 4 °C, and the injection volume was 2 μL. The Orbitrap Exploris 120 mass spectrometer was used for its ability to acquire MS/MS spectra on information-dependent acquisition (IDA) mode in the control of the acquisition software (Xcalibur, Thermo). In this mode, the acquisition software continuously evaluates the full scan MS spectrum. The ESI source conditions were set as following: sheath gas flow rate as 50 Arb, Aux gas flow rate as 15 Arb, capillary temperature 320 °C, full MS resolution as 60,000 eV, MS/MS resolution as 15,000 eV, collision energy: SNCE 20/30/40, spray voltage as 3.8 kV (positive) or -3.4 kV (negative), respectively.

### 2.9 Metagenomic sequencing to monitor microbial communities

#### 2.9.1 DNA extraction, library construction, and sequencing

Referencing previous study (Wang, Shi, et al., 2024), Total genomic DNA was extracted from SSP samples using the E.Z.N.A.® Soil DNA Kit (Omega Bio-Tek, Norcross, GA, U.S.) according to the manufacturer’s instructions. The DNA extract was fragmented to an average size of approximately 400 bp using Covaris M220 (Gene Company Limited, China) for paired-end library construction. Paired-end libraries were constructed using NEXTflex™ Rapid DNA-Seq (Bioo Scientific, Austin, TX, USA). Sequencing was performed on the Illumina NovaSeq 6000 platform at BeijingTsingke Biotech Co., Ltd. (Beijing, China). Raw reads from metagenome sequencing were processed to generate clean reads by removing adaptor sequences, trimming, and eliminating low-quality reads (with N bases, a minimum length of 50 bp, and a minimum quality score of 20) using fastp on the Majorbio Cloud Platform. Clean reads were mapped to the *Glycine max* (L.) Merr reference genome using BWA to identify and remove host-originated reads.

#### 2.9.2 Identification of microbial species

High-quality reads were assembled into contigs using MEGAHIT, retaining contigs longer than 500 bp as the final assembly result. Open reading frames (ORFs) in contigs were identified using Prodigal, and ORFs longer than 200 bp were translated into amino acid sequences using the NCBI translation table. A non-redundant gene catalog was constructed using CD-HIT with 95% sequence identity and 90% coverage. Reads after QC were mapped to the non-redundant gene catalog with 95% identity using Bowtie2, and gene abundance in each sample was evaluated.

### 2.10 Statistical analysis

Data analysis was performed using GraphPad Prism v10.2.0 and R language v3.3.1. Each experiment was conducted independently in triplicate, with results expressed as the mean ± standard deviation. Spearman's correlation coefficient was used for correlation analysis, and visualizations were generated using the Metware Cloud tool (https://cloud.metware.cn/). The analysis of intergroup differences was conducted using one-way analysis of variance (ANOVA). Statistical significance was set at a *p*-value of < 0.05.

## 3 Results and discussion

### 3.1 Analysis of amino acid composition in SSP with different enhanced fermentation strategies

**Fig. 1:**
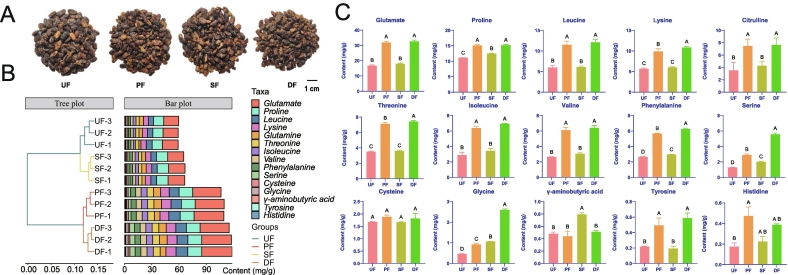
Effects of different enhanced fermentation strategies on the 15 amino acids in SSP. (A) Images of SSP samples with different enhanced fermentation; (B) Stacked bar graph of the 15 amino acids in SSP samples with different enhanced fermentation; (C) Bar graph of the 15 amino acids in SSP samples with different enhanced fermentation.

No significant differences in the appearance of SSP were observed following enhanced fermentation with *A. oryzae* and *B. subtilis* (Fig. 1A). Amino acids, primarily resulting from protein hydrolysis, play a pivotal role in flavor development in soybean products (Nishimura & Abe, 2025). To evaluate the impact of different enhanced fermentation strategies on the amino acid profile of SSP, a total of 15 amino acids were identified across all samples (Fig. 1B). glutamate (16.79-32.71 mg/g) exhibited the highest concentration in all groups, followed by proline (11.18-15.23 mg/g), leucine (6.01-12.13 mg/g), and lysine (5.70-10.87 mg/g). glutamate, known for its umami flavor, enhances taste and accelerates the Maillard reaction, thereby contributing to the formation of additional flavor compounds (Huang et al., 2023). Compared to the UF group, the PF group significantly increased the levels of 13 amino acids, excluding GABA and cysteine (*p* < 0.05). The SF group significantly elevated the content of serine, glycine, GABA, proline, and phenylalanine (*p* < 0.05), while the DF group raised the levels of 12 amino acids, excluding histidine, GABA, and cysteine (*p* < 0.05) (Fig. 1C). Notably, GABA, which positively influences neurological health (Guan et al., 2025; Li and Seugnet, 2025), was significantly enhanced in the SF group, likely due to the role of *B. subtilis* in its conversion (Gao et al., 2020).

Amino acids also contribute to various flavor profiles, including aroma (tyrosine, phenylalanine), sweetness (serine, threonine, proline), umami (glutamate, glycine, lysine), and bitterness (valine, isoleucine, leucine, histidine) (Yang, Shi, et al., 2020; Zhang et al., 2023). Based on the TAV > 1 principle, threonine (1.35), proline (3.73), valine (6.65), isoleucine (3.23), leucine (3.16), phenylalanine (2.97), glutamate (55.96), and lysine (11.40) were identified as key flavor amino acids in the UF group, produced during natural fermentation of SSP (Table S3). Enhanced fermentation significantly promoted the conversion of these amino acids, with the DF group showing the most pronounced effect. Interestingly, serine and histidine exhibited TAV values exceeding 1 after enhanced fermentation in the PF (1.93, 2.38), SF (1.35, 1.13), and UF (3.72, 1.94) groups, while glycine showed a significant increase in the DF group (2.00). These results are consistent with findings reported by Chai et al. (2017).

Collectively, these findings suggest that *A. oryzae* is fundamental for liberating amino acids from SSP, and the addition of *B. subtilis* significantly boosts this process, resulting in the highest yield in DF, followed by PF, and then SF. Though the enhancement achieved by SF alone was less pronounced compared to that of DF and PF. The increased levels of free amino acids (Fig. 1B) suggest a more significant role of *A. oryzae* than *B. subtilis* in amino acid production. This functional disparity is likely attributable to differences in proteolytic activity between the two species. While *A. oryzae* acts as the primary driver of amino acid liberation through its abundant secretion of neutral and acidic proteases (Hu et al., 2020; Zhu et al., 2017), it serves as a rich source of such enzymes. Importantly, proteases produced by *A. oryzae* exhibit superior stability across diverse environmental conditions. (Gao et al., 2019; Salihi et al., 2017). This enhanced enzymatic stability may be a significant factor contributing to the elevated amino acid content observed upon the introduction of *A. oryzae*. On the other hand, owing to its superior environmental adaptability and broad pH tolerance, *B. subtilis* compensates for alkaline protease insufficiency (Su et al., 2020; Sun et al., 2023), This functional synergy—where *A. oryzae* provides foundational neutral/acidic hydrolysis and *B. subtilis* supplements alkaline proteolysis—collectively enables the complex amino acid profile.

### 3.2 Analysis of flavor substances in SSP with different enhanced fermentation strategies

**Fig. 2:**
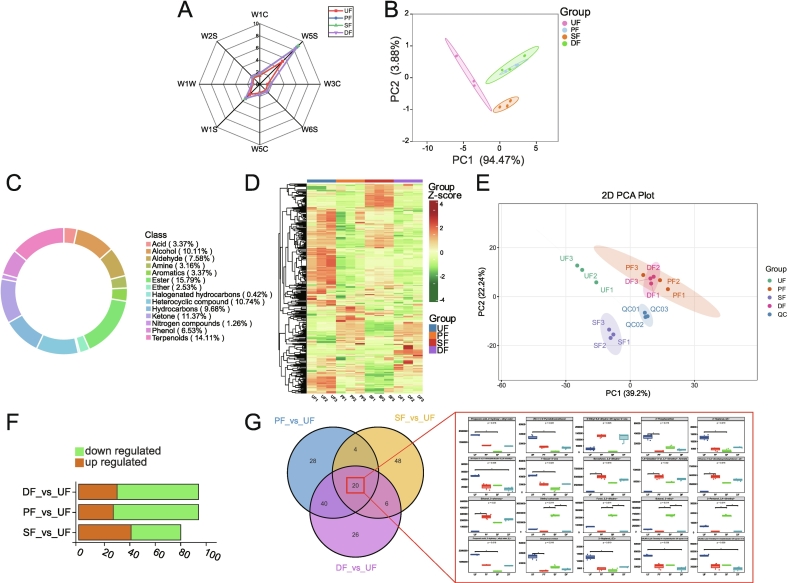
Analysis of flavor substances in SSP with different enhanced fermentation strategies. (A) Radar chart of electronic nose analysis for SSP with different enhanced fermentation strategies; (B) PCA analysis of electronic nose results for SSP with different enhanced fermentation strategies; (C) Classification pie chart of volatile components in SSP with different enhanced fermentation strategies; (D) Heatmap of volatile components in SSP with different enhanced fermentation strategies; (E) PCA plot of volatile components in SSP with different enhanced fermentation strategies; (F) Stacked bar chart of differential volatile components in SSP with different enhanced fermentation strategies; (G) Venn diagram and box plot of shared metabolites among differential volatile components in SSP with different enhanced fermentation strategies.

To evaluate the impact of enhanced fermentation on SSP flavor, E-nose testing was conducted. The results indicated no significant change in the primary aroma components of SSP. The aroma profile remained dominated by W5S sensor (Fig. 2A), suggesting that nitrogen oxides are the primary aroma components in SSP (Wang, Li, et al., 2023), consistent with previous studies (Guo et al., 2024). Principal component analysis (PCA) was then performed to provide a comprehensive overview of the aroma profile in SSP with different enhanced fermentation strategies (Fig. 2B). The results demonstrated that after treatment with various enhancement methods, the overall aroma of SSP significantly deviated from that of the UF group, with various flavor substances undergoing changes and contributing to a more complex aroma; critically, the flavor profiles of PF and DF exhibited remarkable similarity while distinctly differing from SF. This convergence likely stems from the comprehensive enzymatic portfolio of *A. oryzae*—including proteases and amylases—which generates essential aroma precursors and establishes a robust biochemical foundation for subsequent fermentation (Carroll et al., 2017). Consequently, the involvement of *A. oryzae* profoundly reshapes the volatile landscape.

To further explore the effects of enhanced fermentation strategies on the volatile compounds in SSP, HS-SPEM-GC-MS was employed. UF served as the control group, while PF, SF, and DF were the experimental groups. Three comparison groups were designated: UF vs. PF, UF vs. SF, and UF vs. DF. A total of 475 metabolites were detected and annotated, including 75 esters, 67 terpenoids, 54 ketones, 51 heterocyclic compounds, 48 alcohols, 46 hydrocarbons, 36 aldehydes, 31 phenols, 16 aromatics, 16 acids, 15 amines, 12 ethers, 6 nitrogen compounds, and 2 halogenated hydrocarbons. Among all identified metabolites, the majority were esters (15.79%), followed by terpenoids (14.11%) (Fig. 2C), which aligns with previous results (Guo et al., 2024). In the heatmap (Fig. 2D), significant transformations in aroma components were observed for PF, SF, and DF compared to UF, with many metabolites showing a marked downregulation after enhanced fermentation, particularly in the PF group. As shown in Fig. 2E, SF, PF, and DF were distinctly separated from UF, further indicating a substantial influence of enhanced fermentation on the flavor substances of SSP; critically, PF and DF exhibited greater similarity to each other than to SF in their flavor profiles, consistent with the trends identified by electronic nose analysis. The QC samples were tightly clustered, demonstrating the reproducibility and reliability of the experiment (Wang, Hu, et al., 2023).

Using the OPLS-DA model (Figure S1), this study compared the metabolite levels of paired samples to assess PF vs. UF (R^2^Y=0.999, Q^2^=0.961), SF vs. UF (R^2^Y=1, Q^2^=0.969), and DF vs. UF (R^2^Y=1, Q^2^=0.974). All samples had Q^2^ values exceeding 0.9, indicating that the OPLS-DA model is reliable and suitable for identifying differential metabolites. Therefore, we set VIP > 1 and the absolute value of log₂FC ≥ 1 as thresholds for screening differential metabolites. The differential metabolites in the various comparison groups are presented in Fig. 2F, highlighting that the three enhanced fermentation strategies induced distinct metabolic changes compared to UF (Figure S2). In the PF vs. UF comparison, heterocyclic compounds were notably downregulated in PF. These compounds, primarily formed through non-enzymatic browning reactions, are significant volatile components in many foods, known for their complex and intense aroma profiles, making them valuable flavor agents (Bork et al., 2024). This suggests that enhanced fermentation involving *A. oryzae* influences the flavor diversity of SSP. In contrast, the DF group exhibited an increase in heterocyclic compounds compared to UF, indicating that post-fermentation enhancement with *B. subtilis* promotes the formation of complex flavors by upregulating heterocyclic metabolites. When *B. subtilis* was applied alone in the post-fermentation stage, alcohols, aldehydes, and ethers emerged as key differential metabolites between SF and UF. These classes of metabolites are key aroma components in many fermented products (Zhang et al., 2025), contributing significantly to their aromatic profiles. Notably, microbial activity facilitates the reaction of fatty acids with alcohols to form ether compounds, with temperature and pH influencing their production (Cho et al., 2020). Ketones, on the other hand, are generated through the oxidation of alcohols (Zhu et al., 2013), typically produced from sugars during fermentation (Genisheva et al., 2014). This complex transformation process, driven by *B. subtilis*, plays a critical role in shaping the flavor of SSP.

To comprehensively elucidate the impact of enhanced fermentation strategies on differential metabolites in SSP. The KEGG enrichment analysis revealed that distinct microbial reinforcement strategies specifically modulated key metabolic pathways. PF significantly enriched the degradation of aromatic compounds pathway (Figure S3A), potentially enhancing product flavor profiles by reducing undesirable flavor precursors (Mäkelä et al., 2015). SF specifically enriched the riboflavin biosynthesis pathway (Figure S3B), suggesting its potential as a biofortification strategy to improve the nutritional value of fermented products (Saedisomeolia & Ashoori, 2018). Notably, DF uniquely enriched sesquiterpenoid and triterpenoid biosynthesis pathways (Figure S3C). Given that terpenoids typically exhibit significant bioactivities (Chen and Liu, 2017).

### 3.3 Screening of key flavor substances in SSP with different enhanced fermentation strategies

The Venn diagram (Fig. 2G) illustrates 172 differential metabolites across the three comparison groups, with 20 metabolites common to all groups. Among these 20 metabolites, 11 were found at higher concentrations in the UF group, suggesting that all three enhanced fermentation strategies reduce the levels of volatile metabolic components in unfermented SSP. Notably, 2-phenoxy-ethanol, is used as a preservative and insecticide in cosmetics and vaccines (Germinara et al., 2024; Ranade et al., 2023), showed elevated concentrations across all enhanced fermentation methods. SSP, with its distinct flavor profile, has attracted significant attention (Guo et al., 2024).

Following the rOAV ≥ 1 criterion, 51 key flavor metabolites were identified from the 172 differential metabolites (Table S4). Among these, 2,3-dihydro-benzofuran, (rOAV, 258.29), germacrene D (rOAV, 39.54), and 3-furanmethanol (rOAV: 4.45) were most concentrated in PF; 1-hexen-3-ol (rOAV, 1055.49), 3-ethyl-phenol (rOAV, 92.65), and 2-ethyl-butanal (rOAV, 41.98) were highest in SF; while 2-ethyl-3,5-dimethyl-pyrazine (rOAV, 18370.63), (E, Z)-2,6-nonadienal (rOAV, 3525.67), and allyl methyl sulfide (rOAV, 4136.52) were most concentrated in DF.

To further explore the flavor contributions of these differential metabolites, a flavor association network was employed to analyze the metabolites and their sensory flavor annotations, focusing on the top 10 sensory flavors with the highest annotation counts for network diagram creation. The network analysis revealed that, compared to UF, PF exhibited significantly downregulated levels of 2-ethyl-butanal (rOAV, UF: 6.87, PF: 0.98) and 1-octen-3-ol (rOAV, UF: 254.81, PF: 82.76), leading to in a reduction in sweet and fruity flavors (Figure S3D). This downregulation may be attributed to the metabolic activity of *A. oryzae*, which likely reduces the accumulation of aldehyde and alcohol flavor compounds derived from lipid oxidative metabolic pathways. Potential mechanisms include the action of specific oxidoreductases or preferential utilization of precursor substrates (Akakabe et al., 2005; Fu et al., 2023; Lee et al., 2020). SF demonstrated a significant upregulation of 2-ethyl-butanal (rOAV, UF: 6.87, SF: 41.98) and Z-4-decenoic acid methyl ester (rOAV, UF: 3.57, SF: 7.49), which enhanced green and fruity flavors (Figure S3E), This elevation likely originates from the activity of *B. subtilis*, which may employ efficient fatty acid β-oxidation and esterification pathways to synthesize specific aldehydes and esters **(**Fujihashi et al., 2014**;** Mi et al., 2025**)**. In DF, the downregulation of 1-octen-3-ol (rOAV, UF: 254.81, DF: 85.53) and (E, E)-2,4-nonadienal (rOAV, UF: 97.77, DF: 48.45) resulted in the loss of some fruity and fatty flavors (Figure S3F), This phenomenon may arise from complex interactions between co-existing *A. oryzae* and *B. subtilis* during fermentation. For example, synergistic transformation of substrates may jointly regulate end products in related fatty acid oxidation pathways **(**Hyeon et al., 2020**;** Rychen et al., 2018**)**.

### 3.4 Analysis of isoflavone components in SSP with different enhanced fermentation strategies

**Fig. 3:**
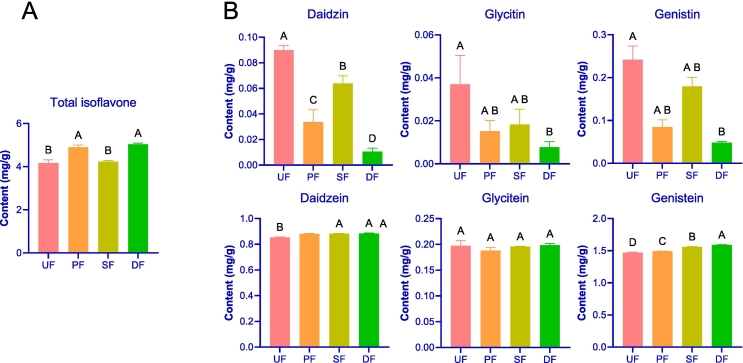
Changes in isoflavones in SSP with different enhanced fermentation strategies. (A) Bar chart of total isoflavones in SSP with different fermentation strategies; (B) Bar chart of six isoflavones in SSP with different fermentation strategies.

Isoflavones are key bioactive compounds in SSP, serving as key indicators of its medicinal value (Yang, Sun, et al., 2020). This study demonstrates that the total isoflavone content in PF (4.89 mg/g) and DF (5.03 mg/g) significantly exceeds that in UF (4.17 mg/g) (*p* < 0.05) (Fig. 3A). Absolute quantification of six key isoflavones in SSP (Fig. 3B) genistein revealed as the predominant isoflavone, with the highest content across all groups (1.47-1.59 mg/g), followed by daidzein (0.85-0.89 mg/g). Daidzein is known for its potent anti-inflammatory and immune-regulating effects (Goleij et al., 2024), while genistein (Nisha, & Paramanik, V., 2024) offers significant neuroprotective benefits. These two isoflavones are essential markers for SSP quality control (Chinese Pharmacopoeia Commission, 2020). Additionally, levels of genistein and daidzein levels in PF, SF, and DF were significantly higher than in UF (*p* < 0.05), with DF showing the highest content, while glycitein levels remained relatively unchanged (Fig. 3B). Daidzin, genistin, and glycitin can be converted into their corresponding aglycones—daidzein, genistein, and glycitein—enhancing their biological activity and bioavailability (Chen et al., 2012; Lin et al., 2021). Quantification of daidzin, genistin, and glycitin provides an alternative measure of isoflavone conversion in SSP. The results indicate that consumption of daidzin was significantly higher in the enhanced fermentation groups compared to UF, with DF exhibiting the most pronounced consumption. In contrast, the levels of genistin and glycitin decreased in DF, PF, and SF relative to UF, with DF showing a significantly lower content (Fig. 3B).

These changes in isoflavone glycoside content and consumption suggest that PF, SF, and DF notably improve SSP quality, with the dual-enhanced fermentation involving *A. oryzae* and *B. subtilis* playing a key role in increasing isoflavone content, followed by PF, and then SF. These isoflavones contribute positively to the antioxidant properties of SSP (Kumar et al., 2023). It is noteworthy that β-glucosidase plays a crucial role in the conversion of isoflavone glycosides to their aglycones (Hati et al., 2015). Both *A. oryzae* and *B. subtilis* exhibit high β-glucosidase activity (Asha et al., 2015; Qin et al., 2011). Previous studies have demonstrated that co-fermentation with *A. oryzae* and *Bacillus amyloliquefaciens* can promote increased β-glucosidase activity (Gil et al., 2018). This study further supports that *A. oryzae* and *B. subtilis* likely enhance the conversion of isoflavone glycosides to aglycones by increasing β-glucosidase activity through microbial interactions.

### 3.5 Comparison of bioactivity in SSP with different enhanced fermentation strategies

**Fig. 4:**
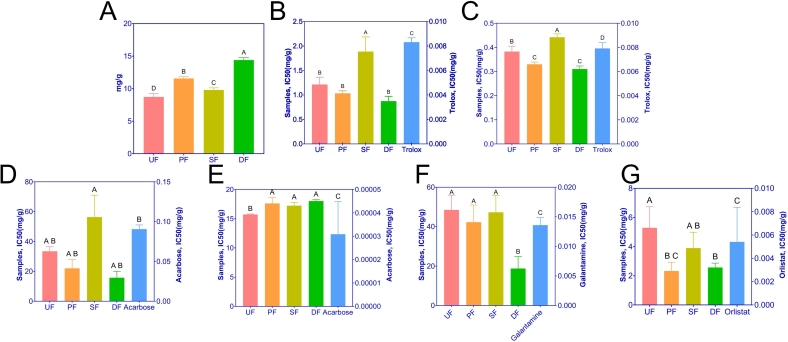
Effects of different enhanced fermentation strategies on antioxidant activity and inhibition rates of four enzymes in SSP. (A) Bar chart of FRAP activity; (B) Bar chart of DPPH activity IC50 values; (C) Bar chart of ABTS activity IC50 values; (D) Bar chart of α-amylase inhibition IC50 values; (E) Bar chart of α-glucosidase inhibition IC50 values; (F) Bar chart of AChE inhibition IC50 values; (G) Bar chart of pancreatic lipase inhibition IC50 values. Means with different superscript letters within the same graph indicate significant statistical differences, *p*-value ＜ 0.05. The left y-axis represents the IC50 values of the samples, the right y-axis represents the IC50 values of positive control drugs.

This study established a dose-dependent relationship between antioxidant activity, AChE inhibition, and the activities of α-amylase, α-glucosidase, and pancreatic lipase in SSP samples at varying concentrations (Figure S4). The overall bioactivity of SSP was assessed by determining the concentration required to reduce enzyme activity and oxidative free radicals by 50% (IC50). In terms of antioxidant capacity, total antioxidant activity measured by FRAP was significantly higher in PF, SF, and DF compared to UF (*p* < 0.05), with DF exhibiting a notably greater capacity than PF and SF (*p* < 0.05) (Fig. 4A). However, the trends observed in the ABTS and DPPH assays did not align with those in FRAP. In the DPPH assay, IC50 values for SF increased significantly, while the other groups showed a decreasing trend. A similar pattern was observed in the ABTS assay (Fig. 4B-C). Interestingly, after inoculation with *B. subtilis*, the antioxidant capacity appeared to diminish, contrary to prior expectations (Dai et al., 2024). This may be due to a reduced abundance of microorganisms involved in antioxidant component production. For α-amylase inhibition, IC50 values decreased following various enhanced fermentation treatments, except in SF, where an increase was observed (Fig. 4D), though no significant differences were noted. In α-glucosidase inhibition experiments, IC50 values for PF, SF, and DF were significantly higher than UF (*p* < 0.05) (Fig. 4E), indicating that the targeted enhanced fermentation strategies may reduce the hypoglycemic properties of SSP. In contrast, the IC50 for AChE inhibition was significantly lower in DF compared to the other groups (*p* < 0.05) (Fig. 4F), suggesting a potential benefit of double-enhanced fermentation. Similarly, for pancreatic lipase activity, the IC50 values for PF and DF were significantly lower than those of UF (*p* < 0.05), whereas SF showed a non-significant decrease (Fig. 4G). Despite these trends, all IC50 values were significantly higher than the positive control (Table S5). This may be due to the complex mixture of active compounds in SSP, where only a small fraction of the sample (Spínola et al., 2020) contributes to its bioactivity. Collectively, in terms of bioactivity, DF exhibited the highest activity, followed by PF and SF, primarily due to the synergistic interaction between *A. oryzae* and *B. subtilis*. These results underscore the importance of conducting comprehensive compositional analyses of SSP samples subjected to different enhanced fermentation strategies.

SSP not only provides a unique flavor but also offers substantial medicinal benefits (Wang et al., 2024). Its health-promoting properties, including anti-psychotic effects, blood glucose regulation, lipid-lowering capabilities, and antioxidant activity, have generated significant interest (Chen et al., 2021; Ge et al., 2006; Liu, Huang, et al., 2023; Luo et al., 2021; Wang, Cheng, et al., 2024). α-amylase and α-glucosidase are key enzymes involved in the breakdown of dietary carbohydrates into glucose. Inhibiting these enzymes serves as a therapeutic strategy for managing diabetes, as it slows carbohydrate digestion and reduces glucose absorption in the intestine (Spínola et al., 2020; Zheng et al., 2024). Obesity, which induces insulin resistance in glucose-sensitive tissues, is a major contributor to hyperglycemia (Kwon et al., 2018). Pancreatic lipase, an enzyme responsible for the hydrolysis of dietary lipids, can be inhibited to reduce fat absorption, supporting weight loss efforts (Hao et al., 2024; Lin et al., 2025). Furthermore, AChE and its inhibitors are integral to the early diagnosis and treatment of Alzheimer's disease (Cai et al., 2025). The antioxidant properties of foods are also beneficial in mitigating conditions such as Alzheimer's (Samanta et al., 2024), hyperlipidemia (Nazir et al., 2024), and hyperglycemia (Elisabetta Maccarronello et al., 2024). *In vitro* antioxidant assays, such as ABTS, DPPH, and FRAP, measure the ability of antioxidants to neutralize free radicals or oxidants, thereby reducing oxidative stress. These assays are commonly used to assess the antioxidant capacity of food products (Hu et al., 2025; Wang, Hu, et al., 2023; Wang et al., 2024; Zhao et al., 2024).

### 3.6 Functional component analysis of SSP with different enhanced fermentation strategies

**Fig. 5:**
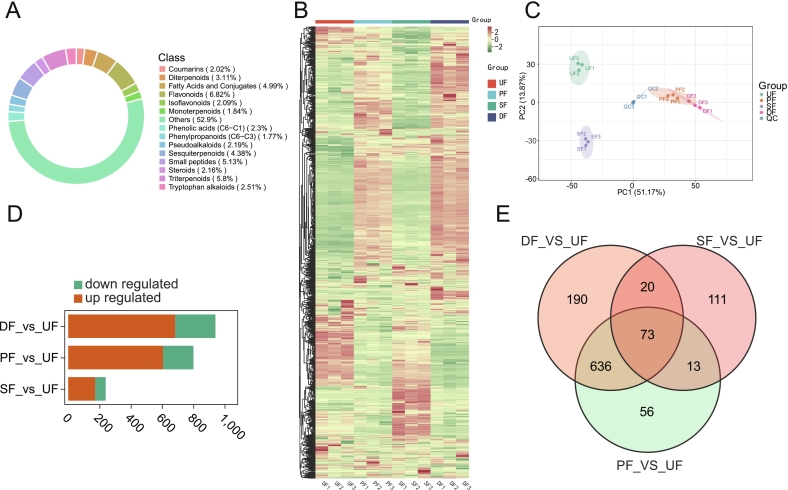
Functional component analysis of SSP with different enhanced fermentation strategies. (A) Classification pie chart of non-volatile components in SSP with different enhanced fermentation strategies; (B) Heatmap of non-volatile components in SSP with different enhanced fermentation strategies; (C) PCA plot of non-volatile components in SSP with different enhanced fermentation strategies; (D) Stacked bar chart of differential non-volatile components in SSP with different enhanced fermentation strategies; (E) Venn diagram of differential non-volatile components in SSP with different enhanced fermentation strategies.

To comprehensively assess the impact of enhanced fermentation on the functional components of SSP, LC-MS/MS was employed, with UF serving as the control group and PF, SF, and DF as experimental groups. Three comparison groups were established: UF vs. PF, UF vs. SF, and UF vs. DF. A total of 2828 metabolites were detected and annotated, including 193 flavonoids, 164 triterpenoids, 145 small peptides, 141 fatty acids and conjugates, 124 sesquiterpenoids, 88 diterpenoids, 71 tryptophan alkaloids, 65 phenolic acids (C6-C1), 62 pseudoalkaloids, 61 steroids, 59 isoflavonoids, 57 coumarins, 52 monoterpenoids, 50 phenylpropanoids (C6-C3), and 1496 others. The predominant classes of metabolites were flavonoids (6.82%), triterpenoids (5.80%), and small peptides (5.13%) (Fig. 5A). Heatmap analysis (Fig. 5B) revealed significant transformations in the component profiles of PF, SF, and DF compared to UF, with a notable increase in metabolite content, particularly in DF. To further investigate the metabolic differences between UF and the other groups, PCA was performed. As shown in Fig. 5C, SF, PF, and DF were distinctly separated from UF, with SF additionally showing clear separation from both PF and DF groups. This confirms that enhanced fermentation significantly affects the non-volatile components of SSP, where PF and DF also differ distinctly from SF. Such divergence likely arises from the abundant β-glucosidases in *A. oryzae* (Watanabe et al., 2016), which catalyze the formation of diverse flavonoid metabolites during fermentation—markedly contrasting with the limited biotransformation capacity of *B. subtilis* monocultures. The QC samples clustered closely together, underscoring the reproducibility and reliability of the experiment.

Using the OPLS-DA model (Figure S5), metabolite content was compared pairwise to assess PF vs. UF (R^2^Y = 1, Q^2^ = 0.977), SF vs. UF (R^2^Y = 1, Q^2^ = 0.926), and DF vs. UF (R^2^Y = 1, Q^2^ = 0.980). All samples had Q^2^ values exceeding 0.9, confirming the reliability of the OPLS-DA model for identifying differential metabolites. Therefore, using VIP > 1 and the absolute value of log_2_FC ≥ 1 as thresholds for differential metabolite selection, 1099 metabolites were found to differ in one or more comparisons. Specifically, compared to UF, PF had 778 differential metabolites (584 upregulated); SF had 217 differential metabolites (150 upregulated); and DF had 919 differential metabolites (661 upregulated) (Fig. 5D). Notably, the number of upregulated metabolites exceeded that of downregulated ones in all comparisons, confirming an increase in many metabolites in SSP after enhanced fermentation. DF had the highest number of upregulated metabolites, significantly affecting SSP quality. The Venn diagram (Fig. 5E) identified 73 differential metabolites across the PF vs. UF, SF vs. UF, and DF vs. UF comparisons. Notably, 13 metabolites, including isoferulic acid and ferulate, were consistently upregulated in all enhanced fermentation groups. Isoferulic acid has been proposed to protect renal and podocyte function in diabetic nephropathy *via* the CXCL12/CXCR4 pathway (Liu et al., 2025), while ferulate regulates lipid metabolism through the AMPK/ACC and PI3K/AKT pathways, improving liver tissue damage and lipid metabolic disorders (Lu & Wang, 2025). These findings suggest that the increased content of specific components resulting from enhanced fermentation likely contributes to the observed changes in SSP activity.

KEGG pathway analysis (Figure S6) further revealed the key metabolic pathways affected by different enhanced fermentation strategies. Enrichment analysis of differential metabolites identified lysine biosynthesis as a pathway predominantly altered in PF and SF (relative to UF), while flavone and flavonol biosynthesis pathways were most significantly enriched in differential metabolites from and DF (relative to UF). Notably, the prominence indicating that amino acids like lysine enhance the nutritional value of SSP (Wang et al., 2024), while, this specific enrichment of flavonoid biosynthesis pathways, particularly pronounced under dual-enhanced fermentation with *A. oryzae* and *B. subtilis*, suggests a positive impact on the generation of bioactive flavonoids in SSP. Among the differential metabolites, 77 flavonoid components were identified as key functional components associated with enhanced fermentation strategies.

### 3.7 Key functional component screening of SSP with different enhanced fermentation strategies

An in-depth analysis was conducted to examine the relationship between 77 flavonoid components and SSP quality. Redundancy analysis (RDA) indicated a strong contribution of these flavonoids to antioxidant capacity and enzyme inhibition (Figure S7A), with most correlations being significantly positive, except for α-glucosidase inhibition, which exhibited an inverse trend. This suggests a potential antagonistic effect of specific flavonoids on α-glucosidase inhibitory activity. Spearman correlation further confirmed a robust association between flavonoid abundance and both antioxidant performance and enzyme inhibition in SSP (Figure S7B). Applying thresholds of *p* < 0.05 and r > 0.80, 70 flavonoid components were identified as strongly associated with enhanced bioactivity (Figure S7B).

Additionally, 19 flavonoid components were further isolated for their positive contributions (including anti-lipase, anti-α-amylase, and antioxidant activities) (Figure S7C). Compared to UF, the PF group exhibited elevated levels of M77 (eriodictyol 7,3'-dimethyl ether), M63 (3,7,3',4'-tetrahydroxyflavanone), M58 (anthraflavic acid), M51 ((2R,3R)-3,5-dihydroxy-2-(4-hydroxyphenyl)-7-(3-methylbut-2-enoxy)-2,3-dihydrochromen-4-one), M48 (isoxanthohumol), M41 (fisetin), M37 (diosmetin), and M24 (5,7-dihydroxy-2-(4-methoxyphenyl)-6-(3-methylbut-2-enyl)chroman-4-one) (*p* < 0.05). In the SF group, M77, M51, M41, and M37 also showed increased concentrations (*p* < 0.05). The DF group displayed significant enrichment of M77, M75 (5,7-dihydroxy-2-[4-hydroxy-3-[(2S,3R,4S,5R)-3,4,5-trihydroxyoxan-2-yl]oxyphenyl]-3-methoxychromen-4-one), M51, M48, M41, M37, and M24 (*p* < 0.05), indicating that dual-stage enhancement more effectively promotes the accumulation of flavonoids than single-stage strategies.

The elevated level of M58 may be closely related to the release of this flavonoid aglycone through the hydrolysis of flavonoid glycoside derivatives by *A. oryzae* glycoside hydrolases (Nguyen et al., 2018; Seo et al., 2018); M51 often undergoes bio-conversion under microbial action (Zhou et al., 2024), while the involvement of *A. oryzae* appears to reduce this conversion efficiency, leading to its higher levels in PF and DF; M41 is recognized for its antioxidant and neuroprotective properties (Maher, 2024; Yang et al., 2024), however, exploration of the biosynthetic pathway of M41 remains incomplete. This study shows significant enrichment of its content in the presence of *A. oryzae*, which may be closely related to multi-microbial interactions influenced by *A. oryzae*; M37 mitigates oxidative stress (Mei et al., 2022) and contributes to the management of type II diabetes by modulating glucose metabolism (Gong et al., 2021). It usually exists in glycosidic form, and its content significantly increases in the presence of *A. oryzae*, which is closely related to the high activity of β-glucosidase in *A. oryzae* (Handa et al., 2014). However, although *B. subtilis* can enhance the conversion of flavonoids in SSP by *A. oryzae*, its advantage in flavonoid conversion efficiency is not evident when used as the sole fermentation strain. These findings highlight the importance of microbial combinations in modulating key flavonoid components, which in turn affect their anti-lipase, anti-α-amylase, and antioxidant activities.

### 3.8 Microbial community structure analysis of SSP with different enhanced fermentation strategies

A total of 91.67 Gbp of raw sequencing data were generated, with read counts ranging from 47,297,490 to 85,282,030. Following quality filtering, over 97% of reads exhibited a sequencing error rate below 1% (Q20, Table S6), confirming the depth and reliability of the sequencing dataset. The microbial community composition was dominated by bacteria, followed by fungi and archaea. Bacteria accounted for the majority of taxa in UF (80.40%), PF (99.18%), and DF (97.48%), with a comparatively lower abundance in SF (47.06%). Taxonomic classification identified 24 phyla, 317 genera, and 607 species in UF; 17 phyla, 196 genera, and 425 species in PF; 23 phyla, 342 genera, and 680 species in SF; and 17 phyla, 252 genera, and 532 species in DF.

**Fig. 6:**
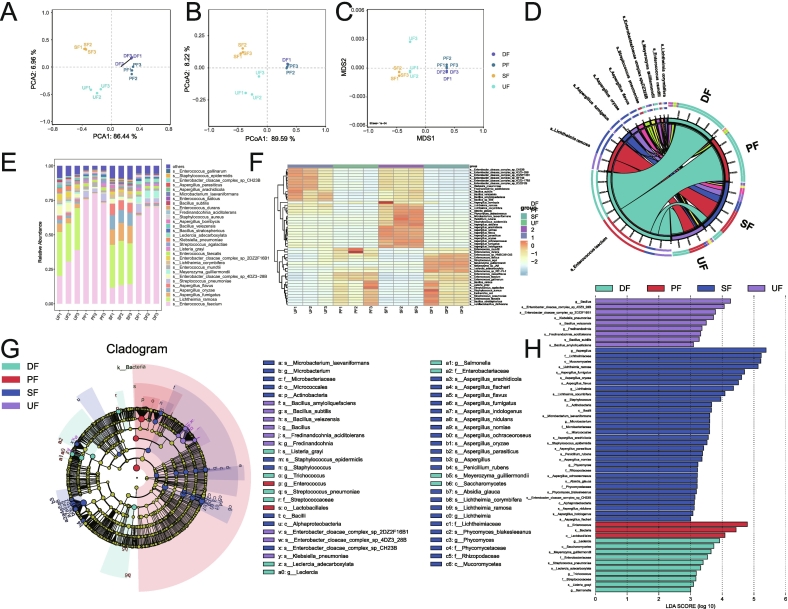
Microbial community characteristics of SSP with different enhanced fermentation strategies. (A) PCA analysis of microbial communities in SSP with different fermentation strategies; (B) PCoA analysis of microbial communities in SSP with different fermentation strategies; (C) NMDS analysis of microbial communities in SSP with different fermentation strategies; (D) Circos plot of the relationships among the top 10 dominant microorganisms in SSP with different fermentation strategies; (E) Stacked bar chart of relative abundance of microorganisms at the species level in SSP with different fermentation strategies; (F) Heatmap of microbial clustering at the species level in SSP with different fermentation strategies; (G) Lefse analysis of microbial communities in SSP with different fermentation strategies; (H) LDA analysis of microbial communities in SSP with different fermentation strategies.

PCA was performed to investigate the overall microbial composition profile in SSP under different enhanced fermentation strategies. The results revealed significant differentiation between PF, SF, and DF compared to UF, with PF and DF showing distinct divergence from SF, indicating substantial shifts in the microbial community following enhanced fermentation. This highlights the differential impacts of these strategies (Fig. 6A). The pronounced divergence of SF from both PF and DF groups may be attributed to antimicrobial cyclic lipopeptides (e.g., surfactin) produced by *B. subtilis*, which significantly restructure the microbial community during SSP fermentation (Kaspar et al., 2019). This observation was further corroborated by PCoA and NMDS analyses (Fig. 6B-C). The relative abundance of the top 10 microorganisms at the species level was analyzed, enabling a comparison of microbial composition across different enhanced fermentation SSP samples (Fig. 6D). At the species level, *Enterococcus faecium* was the dominant microorganism shared among UF (30.91%), PF (76.10%), SF (13.08%), and DF (68.13%). *Enterococcus* has been identified as a key microorganism in SSP fermentation, particularly accumulating during the post-fermentation stage (Wang et al., 2024). As an important member of *Enterococcus*, *E. faecium* has been shown to positively impact the biodegradation of aflatoxin B (Feng et al., 2024). Other microorganisms such as *Lichtheimia ramosa*, *L. corymbifera*, *Enterobacter cloacae complex sp_4DZ3-28B*, and various *Aspergillus* species (e.g., *A. fumigatus*, *A. oryzae*, and *A. flavus*) were notably enriched in UF and SF, while their relative abundance was lower in PF and DF (Fig. 6E). *Lichtheimia* species, common in fermented soybean products (Kim et al., 2024; Luo et al., 2024), have been reported to enhance ABTS radical scavenging activity (*L. ramosa*) and promote the production of flavor amino acids contributing to sweetness and saltiness (*L. corymbifera*). Notably, inoculating *A. oryzae* during pre-fermentation led to a lower relative abundance of *Aspergillus* species in the final SSP product. This observation could be linked to the enzymatic activities of *Aspergillus*, such as α-amylase, β-amylase, protease, and cellulase, which break down and consume various substrates during pre-fermentation (Uwineza et al., 2024), potentially resulting in a nutritional disadvantage for PF and DF during the post-fermentation stage.

Additionally, the pathogenic bacterium *Streptococcus pneumoniae* showed increased relative abundance in PF (4.53%) and DF (4.65%) compared to UF (0.92%), while SF group exhibited a decrease (0.40%). The relationship between inoculated and pathogenic microorganisms requires further investigation. *Meyerozyma guilliermondii* exhibited higher abundance in UF (1.16%) and DF (4.97%), with its aldehyde ketone reductase positively influencing mycotoxin biodegradation (Zhang et al., 2024). *Enterococcus mundtii*, known for its acidification, protein hydrolysis, cellulose degradation, and amylase activities (Nawaz et al., 2019), was relatively abundant in PF (2.31%) and DF (1.49%) (Fig. 6E-F).

Beyond the top 10 dominant microorganisms, 10 additional microorganisms with relative abundances exceeding 1% were identified as dominant in different enhanced fermentation SSPs: *Enterobacter cloacae complex sp_2DZ2F16B*, *Enterococcus faecalis*, *Klebsiella pneumoniae*, *Aspergillus bombycis*, *Leclercia adecarboxylata*, *Streptococcus agalactiae*, *Listeria grayi*, *Bacillus velezensis*, *B. stratosphericus* and *B. stratosphericus* (Table S7). Notably, *B. velezensis* and *B. stratosphericus* are commonly used as functional fermentation strains in various fermented foods, such as tobacco leaves (Zhou et al., 2024) and white shrimp (Gao et al., 2020).

### 3.9 Differential microbial analysis of SSP with different enhanced fermentation strategies

To identify differential microorganisms between enhanced and non-enhanced fermentation SSP, Lefse and LDA analyses were performed. Lefse analysis revealed significant differences in the abundance of the genera *Bacillus*, *Aspergillus*, and *Enterococcus*, suggesting these genera may play a pivotal role in influencing SSP quality under enhanced fermentation conditions (Fig. 6G). Based on an LDA score greater than 3, 29 microorganisms were distinctly classified at the species level, with 7 species showing higher abundance in UF, 18 species in SF, and 4 species in DF (Fig. 6H). Among these, 14 microorganisms were identified as key contributors in different enhanced fermentation SSP due to their relative abundance exceeding 1% and LDA scores of ≥3.

### 3.10 Microbial functional analysis of SSP with different enhanced fermentation strategies

**Fig. 7:**
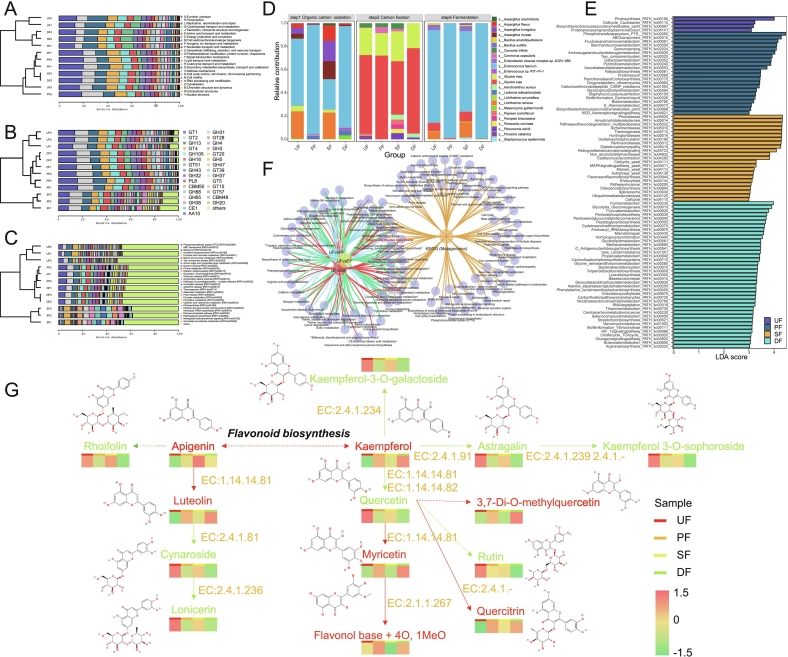
Microbial community functional analysis of SSP with different enhanced fermentation strategies. (A) KEGG database analysis of microorganisms in SSP with different fermentation strategies; (B) CAzy database analysis of microorganisms in SSP with different fermentation strategies; (C) eggNOG database analysis of microorganisms in SSP with different fermentation strategies; (D) Carbon cycle analysis of microorganisms in SSP with different fermentation strategies; (E) Screening of differentially enriched pathways in the KEGG database for microorganisms in SSP with different fermentation strategies; (F) Venn diagram of differentially enriched pathways in the KEGG database and non-volatile component enrichment pathways for microorganisms in SSP with different fermentation strategies; (G) Metabolic profile of flavone and flavonol biosynthesis in microorganisms from SSP with different fermentation strategies.

The relationship between microbial communities and the flavor and functionality of fermented foods is well established. KEGG pathway enrichment analysis revealed that transcription is the dominant metabolic pathway, followed by replication, recombination and repair., carbohydrate transport and metabolism., translation, ribosomal structure and biogenesis., and amino acid transport and metabolism. Notably, carbohydrate transport and metabolism, along with amino acid transport and metabolism, were more abundant in PF and DF, suggesting a positive impact of inoculated *Aspergillus* on carbohydrate and amino acid metabolism (Fig. 7A). Additionally, analysis of key enzymes involved in carbohydrate metabolism, using the CAzy database, revealed significant enrichment of the Glycosyl Transferases family (GT1, GT2, GT4, GT51) and the Glycoside Hydrolases family (GH13, GH105, GH18, GH43, GH32) in PF and DF (Fig. 7B). EggNOG pathway enrichment analysis identified the phosphotransferase system as the most significant metabolic pathway, followed by ABC transporters, ribosome function, oxidative phosphorylation, fructose and mannose metabolism, starch and sucrose metabolism, two-component systems, and amino sugar and nucleotide sugar metabolism. Notably, most of these pathways were more active in DF and PF, except for oxidative phosphorylation, which was more abundant in UF and SF (Fig. 7C). The carbon cycle, crucial for fermented food production, plays a central role in the fermentation process. A deeper understanding of dynamic changes in the carbon cycle can improve the quality and flavor of fermented foods (Lin et al., 2024). In the bar chart, *E. faecium* and *L. ramosa* showed significant dominance in organic carbon oxidation and fermentation (Fig. 7D), highlighting their distinct contributions to the carbon cycling process. Furthermore, LDA analysis revealed 88 differential metabolic pathways across SSPs with different enhanced fermentation strategies (Fig. 7E). Enrichment analysis of these 88 pathways against functional component pathways identified 27 common pathways, with the flavone and flavonol biosynthesis pathway standing out as a key pathway (Fig. 7F).

Based on the KEGG metabolic pathway results, gene functions associated with the formation of flavonoids and flavonols were analyzed (Fig. 7G). Enhanced fermentation significantly promoted the conversion of Apigenin-Luteolin and Kaempferol-Quercetin-Myricetin by upregulating the activity of flavonoid 3',5'-hydroxylase [EC:1.14.14.81], with the most pronounced effect observed in the DF group. This suggests that the functional microorganisms in DF may exhibit higher levels of flavonoid 3',5'-hydroxylase activity. Notably, luteolin has been shown to alleviate diabetes-related neuroinflammation (Moustafa et al., 2023), while myricetin holds potential as a treatment for type II diabetes by improving glucose metabolism and lipid profiles in mouse models (Babotă et al., 2024). Additionally, under the influence of flavonoid O-methyltransferase [EC:2.1.1.267], myricetin can be further converted into a flavonol base + 4O, 1MeO; and under the action of flavonol-3-O-rhamnosyltransferase [EC:2.4.1.-], quercetin can be converted into quercitrin. This conversion enhances solubility in polar solvents, improving absorption (Chen et al., 2022), with DF showing a positive effect on these transformations. Conversely, during non-enhanced fermentation, kaempferol is converted to kaempferol-3-O-galactoside *via* kaempferol 3-O-β-D-galactosyltransferase [EC:2.4.1.234]; it can also be converted into astragalin through flavonol 3-O-glucosyltransferase [EC:2.4.1.91] and further into kaempferol-3-O-sophoroside *via* flavonol-3-O-glucoside/galactoside glucosyltransferase [EC:2.4.1.239]. Luteolin is converted into cynaroside by flavone 7-O-β-glucosyltransferase [EC:2.4.1.81] and subsequently into lonicerin through flavanone 7-O-glucoside 2”-O-β-L-rhamnosyltransferase [EC:2.4.1.236]. Natural fermentation appears to reduce these conversions, likely due to the influence of inoculated *A. oryzae* and *B. subtilis*, which may affect microorganisms harboring these key enzymes (Sui et al., 2024; Zhao et al., 2024).

### 3.11 Microbial-key flavor substances/functional components correlation network analysis of SSP with different enhanced fermentation strategies

**Fig. 8:**
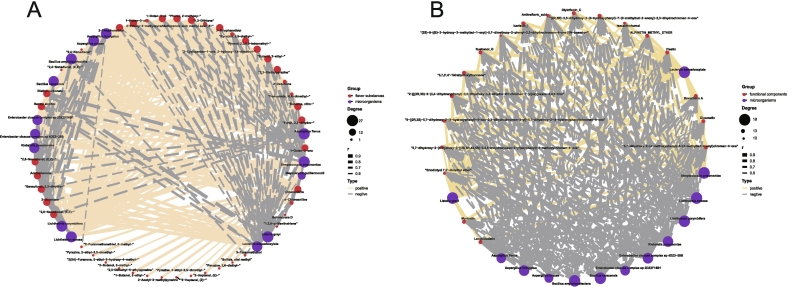
Spearman correlation analysis of microbial-key flavor substances/functional components in SSP with different enhanced fermentation strategies. (A) Correlation analysis between key microorganisms and key flavor substances; (B) Correlation analysis between key microorganisms and key functional components.

Spearman correlation analysis revealed significant associations between the relative abundance of key microorganisms and the presence of essential flavor compounds and functional components. *Aspergillus* species (*A. fumigatus*, *A. oryzae*), *Bacillus* species (*B. amyloliquefaciens*, *B. velezensis*), *Enterobacter* species (*E. cloacae complex sp 2DZ2F16B1*, *E. cloacae complex sp 4DZ3-28B*), and *Lichtheimia* species (*L. corymbifera*, *L. ramosa*) were strongly correlated with a wide array of key flavor compounds. Positive correlations were particularly evident with 1-octen-3-one, diethyl carbonate, 2,3-dihydro-furan, nitrobenzene, N,N-dimethyl-formamide, benzyl alcohol, 2-decanone, 2,3-diethylpyrazine, 2-ethyl-butanal, 2-cyclopenten-1-one, 2-hydroxy-3,4-dimethyl-., 2,3,5,6-tetramethyl-phenol, 2,5-diethyl-pyrazine, 3-thiophenethiol, Z-4-decenoic acid methyl ester, o-chloroaniline, 1,2-dithiane, 2-methoxy-phenol, 1-octen-3-ol, 1-hexen-3-ol, 2-ethoxy-3-methylpyrazine, 2-thiophenethiol, and octanenitrile (Fig. 8A). Notably, 1-octen-3-one, a key flavor component in cooked mushrooms (Guo et al., 2023; Xie et al., 2024), and 3-thiophenethiol, produced through the Maillard reaction, contribute to the characteristic meaty aroma (Cerny & Davidek, 2003; Zhao et al., 2019). While moderate levels of 1-octen-3-ol provide a fatty taste, excessive amounts lead to an undesirable flavor (Dong et al., 2025; Yao et al., 2025). Additionally, 2-ethoxy-3-methylpyrazine, responsible for the nutty aroma in SSP, is also a significant component in the fermentation of Bulang pickled tea (Zhou, Chen, Foo, Cao and Lin, 2024). In contrast, *L. adecarboxylata*, *L. grayi*, and *S. pneumoniae* exhibited strong positive correlations with 19 flavonoid compounds (Fig. 8B). *L. adecarboxylata* mitigates oxidative stress in tomatoes and enhances the levels of bioactive compounds such as flavonoids and phenolics (Haque et al., 2024). While *L. grayi* and *S. pneumoniae*, common environmental microorganisms, harbor a variety of functional enzymes (Li et al., 2025; Silva et al., 2003), their use in fermented foods warrants further safety evaluations.

## 4 Conclusion

This study explored the effects of single and dual-enhanced fermentation using *A. oryzae* and *B. subtilis* on the quality and microbial community of SSP. Multi-omics analyses revealed that dual-enhanced fermentation significantly increased key flavor amino acids and isoflavone conversion rates, while enhancing enzyme inhibition and antioxidant capacity. HS-SPME-GC-MS identified 51 flavor-associated volatiles, and LC-MS/MS with KEGG pathway analysis demonstrated enrichment in flavonoid biosynthesis (*p*<0.05). Spearman correlation highlighted 19 bioactive flavonoids positively linked to core functional microbes (e.g., *A. fumigatus*, *B. amyloliquefaciens*; LDA≥3). Metagenomic sequencing revealed microbial consortia (*Aspergillus*, *Bacillus*, *Enterobacter*) driving flavor and flavonoid accumulation. The dual-enhanced strategy optimized SSP quality by modulating microbial structure, offering a framework for industrial fermentation process improvement.

## CRediT authorship contribution statement

**Bin Wang:** Writing – original draft, Software, Methodology, Investigation, Formal analysis, Data curation. **Tianxing He:** Visualization, Software, Investigation, Data curation. **Yingying Cheng:** Visualization, Software, Data curation. **Hongping Chen:** Data curation, Conceptualization. **Yuan Hu:** Methodology, Investigation. **Youping Liu:** Validation, Software, Resources. **Fu Wang:** Writing – review & editing, Supervision, Project administration, Methodology, Funding acquisition, Conceptualization. **Lin Chen:** Writing – review & editing, Validation, Supervision, Funding acquisition.

## Declaration of competing interest

The authors declare that they have no known competing financial interests or personal relationships that could have appeared to influence the work reported in this paper.

## Data Availability

The original contributions presented in the study are included in this article, further inquiries can be directed to the corresponding authors.
