# Supplementary material for: Targeted enhancement strategies for Sojae Semen Praeparatum: Impact of Aspergillus oryzae and Bacillus subtilis on microbial communities, flavor substances, and functional components
Source: Food Chem X. 2025 Aug 21;30:102931. doi: 10.1016/j.fochx.2025.102931 (PMC12491744; doi:10.1016/j.fochx.2025.102931)
Supplement: Supplementary material 1 — Figs. S1-S7 [file mmc1.docx]

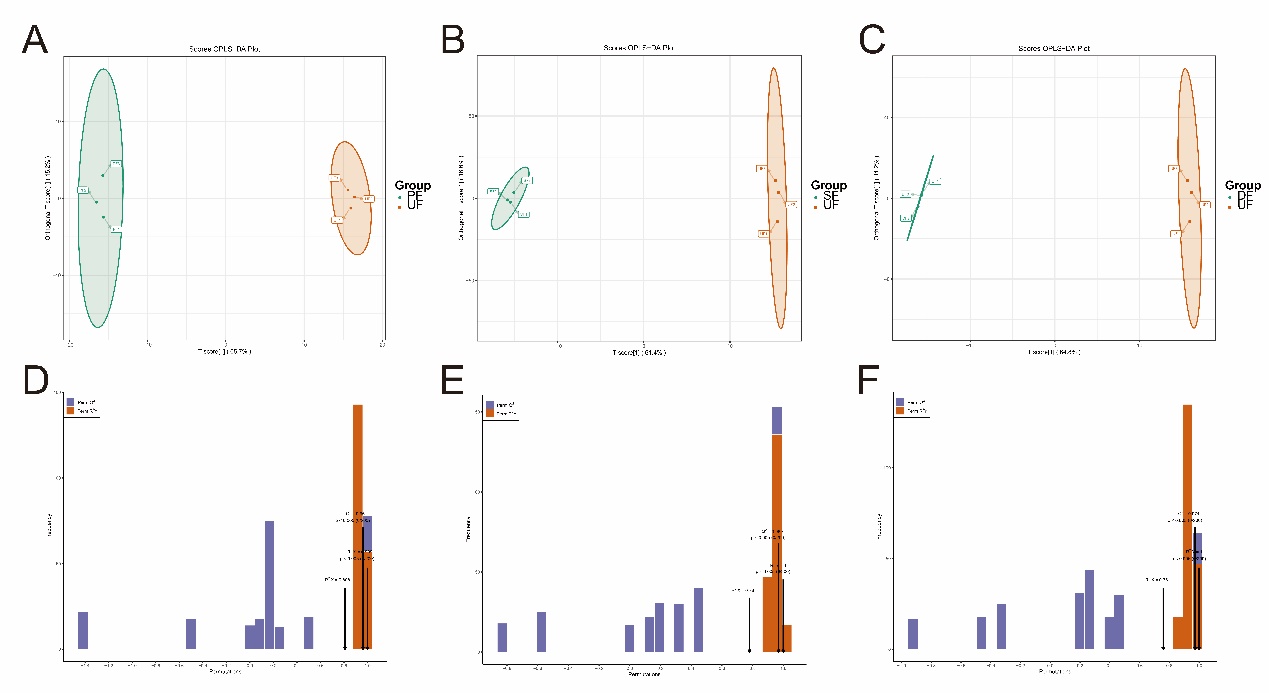


Figure S1. OPLS-DA analysis of volatile components in SSP with different enhanced fermentation strategies. (A) OPLS-DA analysis comparing PF to UF; (B) OPLS-DA analysis comparing SF to UF; (C) OPLS-DA analysis comparing DF to UF; (D) Permutation test plot for the comparison of PF to UF; (E) Permutation test plot for the comparison of SF to UF; (F) Permutation test plot for the comparison of DF to UF.


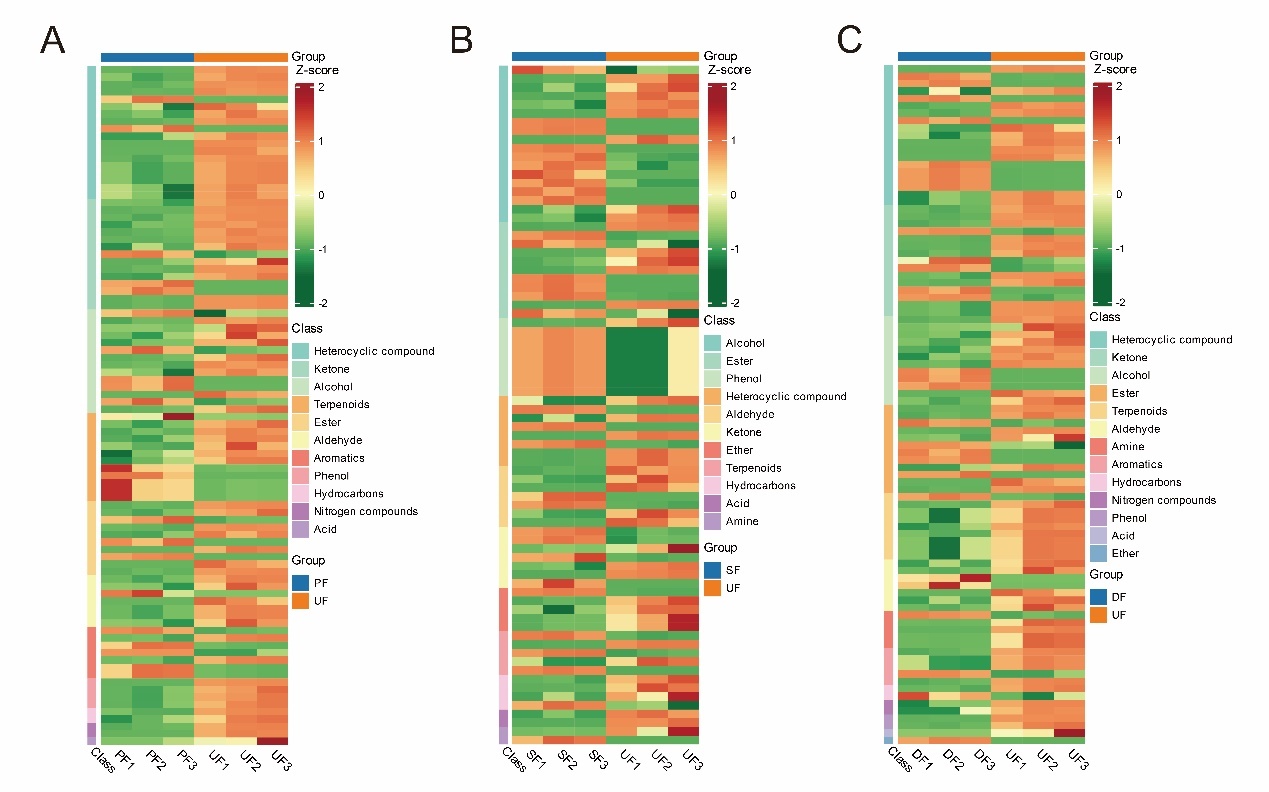


Figure S2. Heatmap analysis of differential metabolites in SSP with different enhanced fermentation strategies. (A) Heatmap of differential metabolites comparing PF to UF; (B) Heatmap of differential metabolites comparing SF to UF; (C) Heatmap of differential metabolites comparing DF to UF.


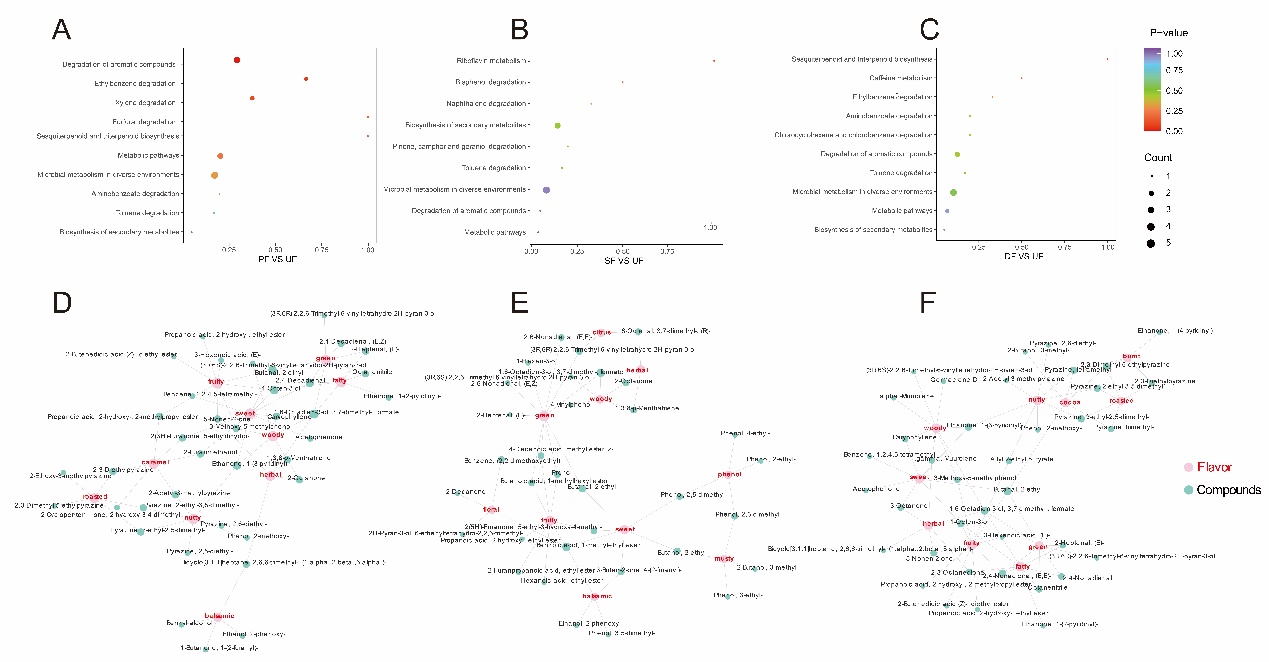


Figure S3. Analysis of flavor networks and KEGG enrichment for differential volatile components in SSP with different enhanced fermentation strategies. (A) KEGG enrichment analysis of differential volatile components comparing PF to UF; (B) KEGG enrichment analysis of differential volatile components comparing SF to UF; (C) KEGG enrichment analysis of differential volatile components comparing DF to UF; (D) Sensory flavor network diagram of differential volatile components comparing PF to UF; (E) Sensory flavor network diagram of differential volatile components comparing SF to UF; (F) Sensory flavor network diagram of differential volatile components comparing DF to UF.


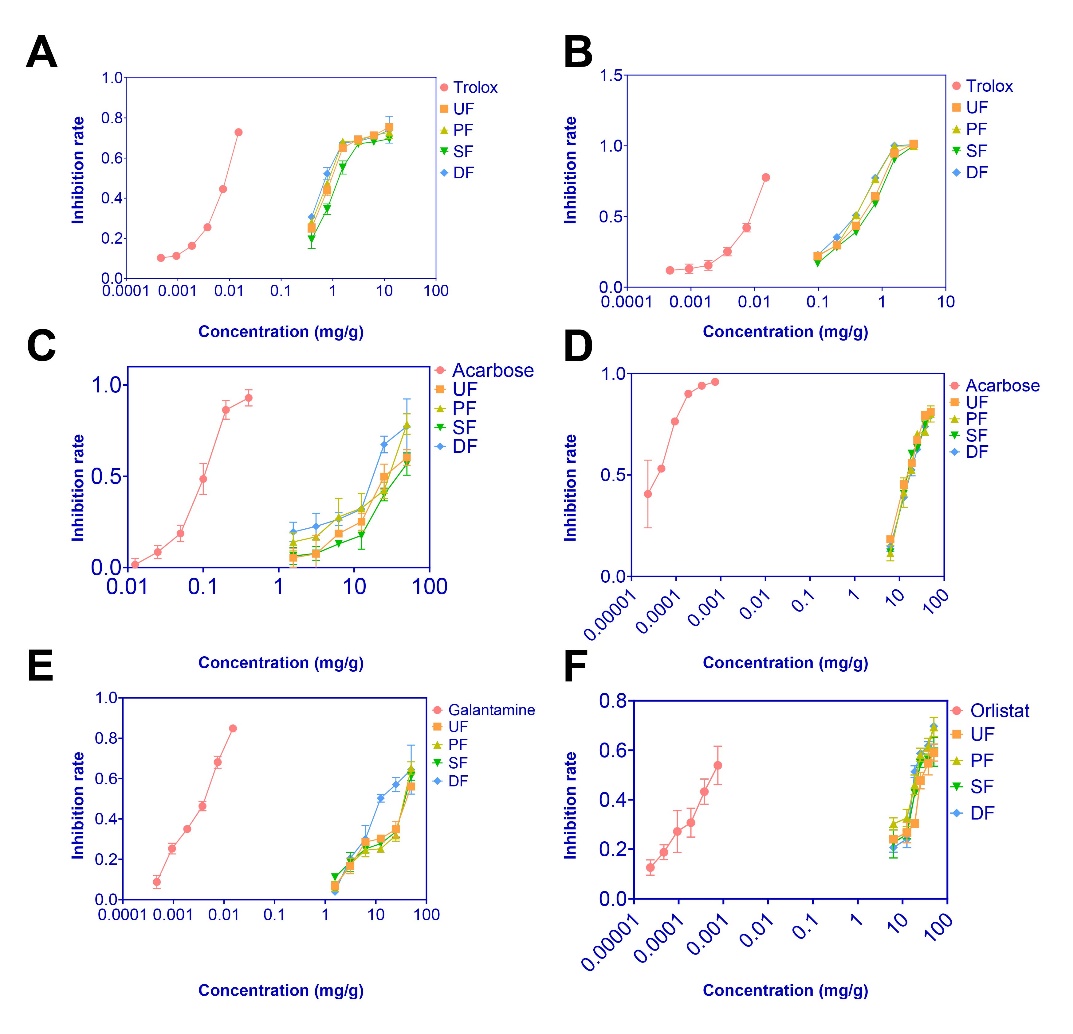


Figure S4. Dose-dependent effects of fermentation strategies on bioactivity in SSP. (A) Line graph of DPPH activity; (B) Line graph of ABTS activity; (C) Line graph of α-amylase inhibition; (D) Line graph of α-glucosidase inhibition; (E) Line graph of acetylcholinesterase inhibition; (F) Line graph of pancreatic lipase inhibition.


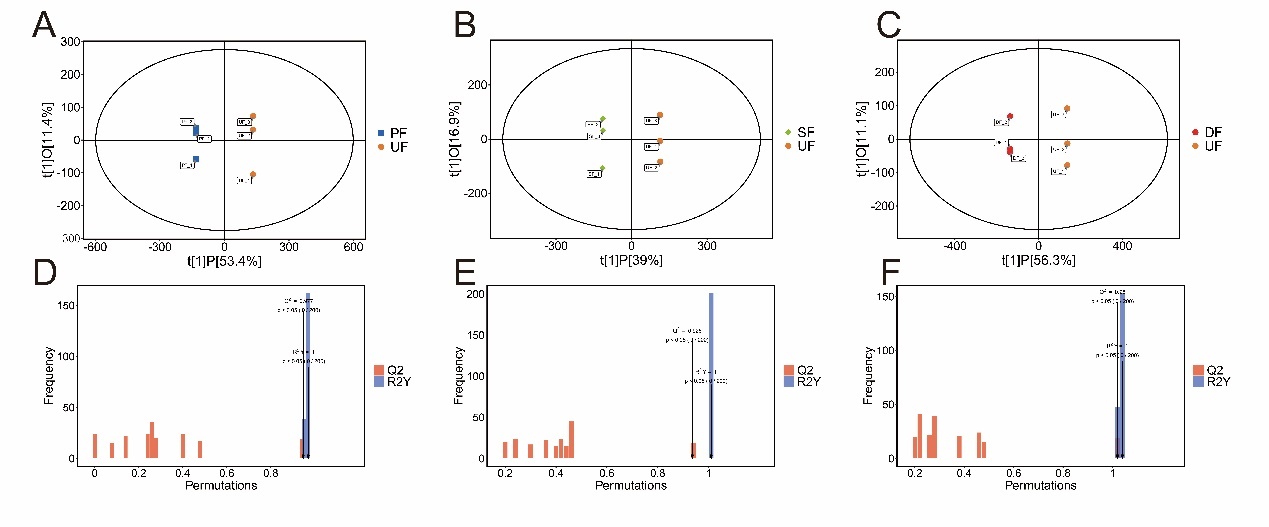


Figure S5. OPLS-DA analysis of non-volatile components in SSP with different enhanced fermentation strategies. (A) OPLS-DA analysis of PF compared to UF; (B) OPLS-DA analysis of SF compared to UF; (C) OPLS-DA analysis of DF compared to UF; (D) Permutation test plot for PF compared to UF; (E) Permutation test plot for SF compared to UF; (F) Permutation test plot for DF compared to UF.


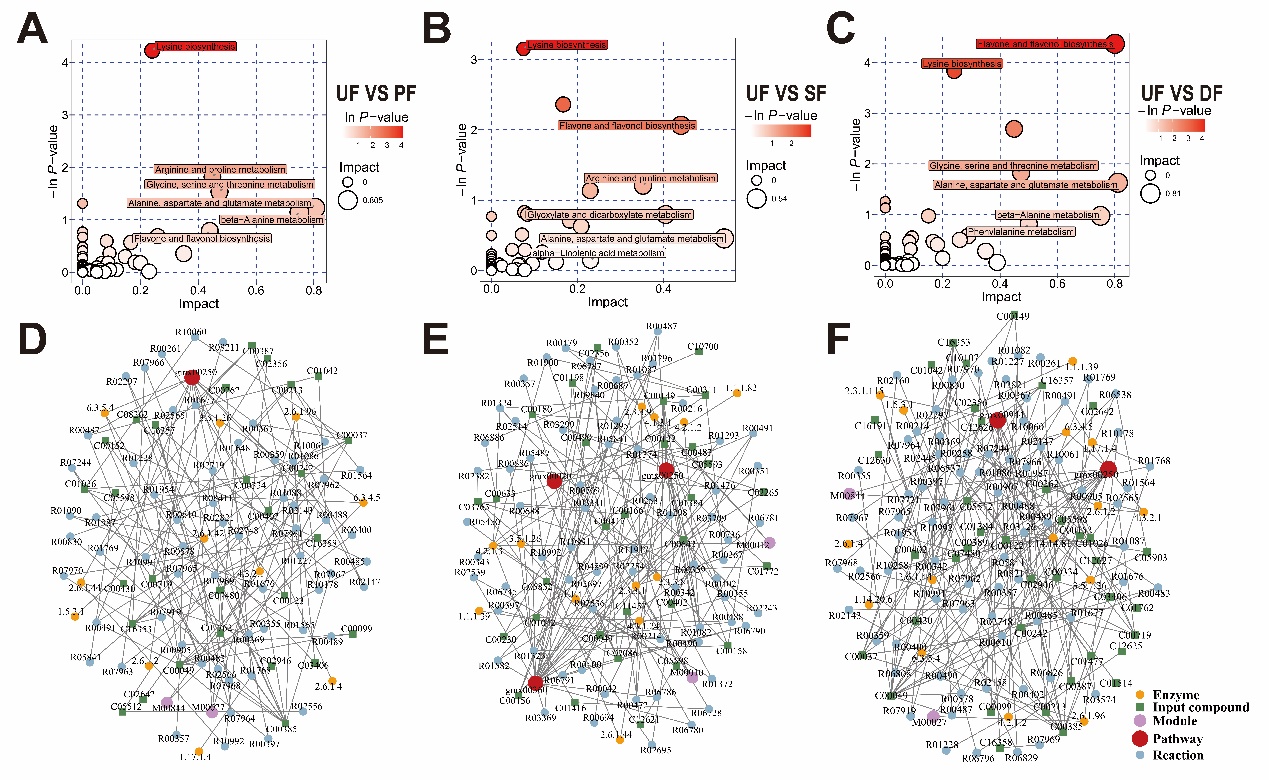


Figure S6. KEGG enrichment analysis of non-volatile components in SSP with different enhanced fermentation strategies. (A) KEGG enrichment analysis of PF compared to UF; (B) KEGG enrichment analysis of SF compared to UF; (C) KEGG enrichment analysis of DF compared to UF; (D) KEGG enrichment network analysis for PF compared to UF; (E) KEGG enrichment network analysis for SF compared to UF; (F) KEGG enrichment network analysis for DF compared to UF.


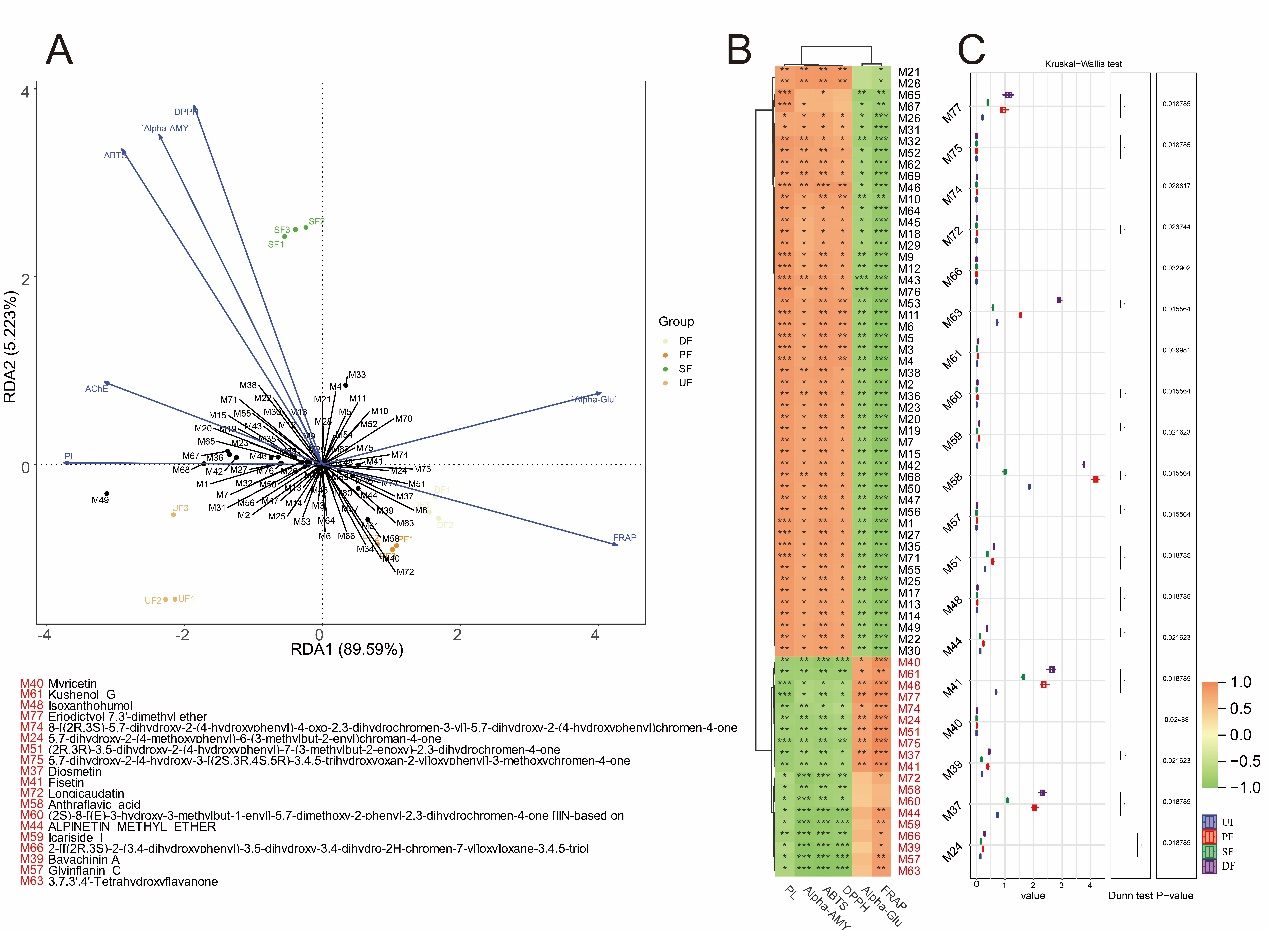


Figure S7. Key functional component analysis of SSP with different enhanced fermentation strategies. (A) RDA analysis of antioxidant and enzyme inhibition activity with flavonoid components; (B) Spearman correlation analysis of antioxidant and enzyme inhibition activity with flavonoid components; (C) Box plot of the contents of 19 key active flavonoid components.
